# Identification of alanine aminotransferase 1 interaction network via iTRAQ-based proteomics in alternating migration, invasion, proliferation and apoptosis of HepG2 cells

**DOI:** 10.18632/aging.204286

**Published:** 2022-09-14

**Authors:** Xiao Fu, Wenyue Zhang, Shiying Li, Ning Ling, Yixuan Yang, Zhang Dazhi

**Affiliations:** 1 Department of Infectious Diseases, Institute for Viral Hepatitis, Key Laboratory of Molecular Biology for Infectious Diseases, The Second Affiliated Hospital of Chongqing Medical University, Chongqing 400010, P.R. China; 2 Department of Paediatrics and Adolescent Medicine, Li Ka Shing Faculty of Medicine, University of Hong Kong, Hong Kong 999077, P.R. China

**Keywords:** alanine aminotransferase 1, hepatocellular carcinoma, mass spectrometry, protein-protein interaction, biological behavior

## Abstract

Objective: To investigate the mechanism of alanine aminotransferase 1 (ALT1) in the progression of HCC, the differentially expressed proteins (DEPs) in the ALT1 interaction network were identified by targeted proteomic analysis.

Methods: Wound healing and transwell assays were conducted to assess the effect of ALT1 on cellular migration and invasion. Cell Counting Kit-8 (CCK-8), colony formation, and flow cytometry assays were performed to identify alterations in proliferation and apoptosis. After coimmunoprecipitation processing, mass spectrometry with iso-baric tags for relative and absolute quantitation was utilized to explore the protein interactions in ALT1 knockdown HepG2 cells.

Results: The results showed that ALT1 knockdown inhibits the migration, invasion, proliferation of HepG2 cells, and promotes apoptosis. A total of 116 DEPs were identified and the bioinformatics analysis suggested that the ALT1-interacting proteins were primarily associated with cellular and metabolic processes. Knockdown of ALT1 in HepG2 cells reduced the expression of Ki67 and epithelial cell adhesion molecule (EP-CAM), while the expression of apoptosis-stimulating protein 2 of p53 (ASPP2) was increased significantly. Suppression of the ALT1 and EP-CAM expression contributed to alterations in epithelial–mesenchymal transition (EMT) -associated markers and matrix metalloproteinases (MMPs). Additionally, inhibition of ALT1 and Ki67 also decreased the expression of apoptosis and proliferation factors. Furthermore, inhibition of ALT1 and ASPP2 also changed the expression of P53, which may be the signaling pathway by which ALT regulates these biological behaviors.

Conclusions: This study indicated that the ALT1 protein interaction network is associated with the biological behaviors of HepG2 cells via the p53 signaling pathway.

## INTRODUCTION

Globally, cancer mortality is greater than the mortality of other diseases, and the cost of treating cancer continues to rise. Hepatocellular carcinoma (HCC) is a category of malignant tumor with a high recurrence rate and ranks fourth in cancer-caused mortality [1]. Key risk factors for HCC progression involve chronic infection with hepatitis B virus (HBV) and hepatitis C virus (HCV) and liver cirrhosis [2]. Moreover, consuming food with aflatoxin-B1, alcoholism dependence, prolonged smoking, and other metabolic diseases, such as type II diabetes, also contribute to the development of HCC [2]. The gender disparity in HCC incidence suggests that approximately 2-8 times as many men develop HCC than women, which may result from the association of sex hormones with the progression of HBV-induced HCC [3, 4]. Tumor metastasis is usually coupled with the aggravation of liver cancer and higher mortality of HCC patients [5]. In addition, deregulated cell proliferation combined with suppressed apoptosis constitutes the minimal common form upon which all neoplastic evolution occurs [6].

Alanine transaminase (ALT) converts alanine into pyruvate in gluconeogenesis, and its two isoforms, ALT1 and ALT2, have different subcellular and tissue distributions [7]. ALT1 is found in the cytosol and is used as a biomarker for liver disease assessment [8]. Studies have suggested that increasing levels of serum ALT are closely related to the incidence of HCC in patients with HBV/HCV [9, 10]. Another study illustrated that the evaluated serum ALT levels are also connected with the incidence of HCC regardless of hepatitis virus negativity [11]. While these previous studies have improved the connection of serum ALT levels with HCC, the effect of cytoplasmic ALT1, before secretion into the serum, on the development and progression of HCC remains unclear.

Isobaric tags for relative and absolute quantitation (iTRAQ) is an established technology to identify DEPs and the protein interactions [12]. Since it can label and analyze eight samples simultaneously, this method can effectively reduce the risk of error during repeated sample testing [13]. In this study, we aimed to use this method to evaluate the variations in the proteome of ALT1 siRNA-treated and control siRNA-treated samples to determine further the proteins participating in the metastasis, proliferation, and apoptosis of HCC.

## MATERIALS AND METHODS

### Antibodies and reagents

Antibodies against ALT1 (A2814), LAMB3 (A13663), HSPB1 (A0240), ASPP2 (A15105), KI67 (A2094), EP-CAM (A19301), MMP2 (A19080) and MMP9 (A2095) were obtained from ABclonal (Wuhan, China). Cleaved caspase 3 (AF7022), Bax (AF0120), and Bcl-2 (AF6139) were obtained from Affinity Biosciences (Cincinnati, OH, USA), and P53 (SC98), P21 (SC53870), CDK4 (SC23896), β-actin (SC58673) and HRP-IgG were obtained from Santa Cruz Biotechnology (Shanghai, China). The cell lines L02, Hep3B, Huh7, and HepG2 were obtained from the Chinese Academy of Sciences (Shanghai, China). iTRAQ eight-plex kits were procured from Applied Biosystems (Thermo Fisher Scientific, Inc., Waltham, MA, USA) and the Transwell kits were obtained from Cell Biolabs (San Diego, CA, USA). Western blot electrophoresis reagents were provided by Bio-Rad Laboratories (Hercules, CA, USA) and the polyvinylidene fluoride (PVDF) membranes were manufactured by GE Healthcare (Chicago, IL, USA). ALT1 (Gene ID: 2875), KI67 (Gene ID: 4288), EP-CAM (Gene ID: 4072), and ASPP2 (Gene ID: 7159)-specific siRNAs and negative control siRNAs were designed by Gene-Pharma (Suzhou, China). Forward/reverse primers used in reverse transcription-quantitative polymerase chain reaction (RT-qPCR) were purchased from TSINGK (Shanghai, China).

### Immunohistochemistry and tissue microarrays

Commercial tissue microarrays containing 50 cases of HCC tissues and matched adjacent non-tumor tissues (Catalog: LV1505, Alenabio Biotechnology, Xi’an, China) were obtained for immunohistochemical (IHC) analysis. The samples were deparaffinized with xylene, rehydrated with an ethanol gradient, and washed with double-distilled H_2_O [14]. The tissues were soaked in 3% H_2_O_2_ for 10 min to quench endogenous peroxidase activity and blocked with BSA for 30 min. The tissue samples were incubated with primary antibodies against ALT1 overnight at 4° C. An EnVision+ System, HRP (DakoCytomation, Glostrup, Denmark) was used to detect the expression of ALT1 under 200x magnification [14].

### Cell culture and ALT1, EP-cam, Ki67, or ASPP2 siRNA transfection

L02, Hep3B, Huh7 and HepG2 cell lines were cultured in a 37° C incubator with 5% CO2 and 95% relative humidity for cell line screening via Western blot analysis. The culture medium was made up of high glucose DMEM (KeyGen Biotech, China) supplemented with 10% fetal bovine serum (FBS) (Excell Bio, Shanghai, China). The HepG2 cells were transfected with ALT1-specific siRNA, EP-CAM-specific siRNA, Ki67-specific siRNA, ASPP2-specific siRNA, or negative control siRNA using Lipofectamine® 2000 (Thermo Fisher Scientific, Inc., Waltham, MA, USA) and Opti-MEM (Gibco; Thermo Fisher Scientific, Inc.). RNA and protein were extracted at 24 h and 48 h, respectively.

### Wound healing and transwell assays

HepG2 cells were transfected with ALT1-siRNA and negative control siRNA in wound healing and Transwell assays, respectively. After the cells had condensed in the 6-wells plate for 48 h, scratches were made along a ruler with a 200 μl pipette tip, and the cellular debris was gently washed out with PBS. The migration was identified by changes in the area for up to 24 h under the microscope at 10 x magnification. The transwell assays were performed with a Cell Migration and Cell Invasion Assay kit. The transfected cells were re-cultured in the upper chambers of 24-well plates with cell medium in the lower chamber. The 8-μm membranes separated the invading/migrating cells, and cell staining buffer and extraction buffer were used to stain and extract the cells beneath the membranes. Twenty minutes after staining, the number of cells that pass through the membrane without or with matrigel was measured by CyQuant GR fluorescent dye (560 nm).

### CCK-8

Transfected cells were plated in 96-well plates at a density of ~2×10^5^ cells per well. 10 μl CCK-8 was added every 12 h and incubated at 37° C for 1 h. The colorimetric results were measured at 450nm. Due to the toxicity of Lipofectamine® 2000 and CCK-8, we narrowed the test interval to every 12h.

### Colony formation assay

The transfected cells were digested with 0.05% trypsin and resuspended in cell culture medium. We replated these cells to 6-well plates and incubated them at 37° C in a 5% CO_2_ incubator for two to three weeks. When the cells showed visible colonies, we stopped the culture and gently washed the cells twice before fixing in 4% PFA for 15 min and dyeing the cells with crystal violet. Colonies containing ≥30 cells were counted. The clone formation of efficiency was defined as the number of formed colonies/the number of seeded cells × 100%. Cells were imaged under a microscope and the stained area was calculated using ImageJ.

### Flow cytometry

After 48h of transfection, cells were centrifuged and resuspended in PBS, divided into labeled EP tubes, and centrifuged at 3000 rpm for 4 min. For cell apoptosis evaluation, the cell pellet was resuspended in PBS. For cell cycle assessment, the cell pellet was resuspended in 70% alcohol. After adding 5 μL of annexin V-FITC and PI (eBiosciences) and avoided light for 15 minutes, apoptosis was detected using the flow cytometric evaluation (Beckman Coulter, CytoFLEX) and FlowJo analysis (Treestar, 10.0.7r2).

### Protein collection CO-IP and iTRAQ labeling

When the cells were approximately 80% confluent, HepG2 cells transfected with ALT1 knockdown siRNA or negative control siRNA were washed three times with PBS and lysed with 500ul-1ml cell lysate buffer. 0.2-2 μg of the primary antibody was added for immunoprecipitation and the lysates were incubated overnight at 4° C with slow shaking. 40 ul of resuspended Protein A+G Agarose was added and shaken slowly at 4° C for 1-3 hours. The samples were centrifuged at 2500 rpm for 5 minutes, and the pellets were washed 5 times with PBS. The supernatant was removed, the pellet resuspended in 20-40 μl 1X SDS-PAGE electrophoresis loading buffer, and the sample centrifuged to the bottom of the tube by instantaneous high-speed centrifugation. The samples were boiled for SDS-PAGE electrophoresis. For the agarose group, samples were accessed through the elimination of the primary antibody. For the IgG group, the samples were obtained by replacing the primary antibodies with IgG antibody. For iTRAQ detection, the eluted proteins were precipitated by acetone at -20° C overnight. According to the iTRAQ manufacturer’s protocol, the proteins were dissolved in dissolution buffer, denatured, cysteine blocked, and then were digested with trypsin. The protein samples were labeled as follows: negative control siRNA transfected protein, 113, 114, and 115 tags; and ALT1 siRNA-treated protein, 116, 117, and 118 tags. The labeled samples were mixed for further analysis.

### Peptide fractionation

The collected labeled protein was soaked in the solution of Pharmalyte (GE Healthcare Life Sciences, Little Chalfont, UK) and urea. The samples were rehydrated on pre-hydrated immobilized pH gradient (IPG) strips (pH 3–10) before isoelectric focusing on an IPGphor system at 68 kV/h. The peptides were extracted from gels by acetonitrile and formic acid incubation and purified on a C18 detection-based DSC-18 SPE column (Supelco, Sigma-Aldrich, Darmstadt Germany). Finally, the peptides were vacuum lyophilized and stored at -20° C before mass spectrometry analysis.

### Mass spectrometry (MS)

Mass spectrometry was performed on a TripleTOF 5600 + LC/MS system (AB Sciex LLC., Framingham, MA, USA). The prepared peptide samples were dissolved in a 2% acetonitrile solution and later analyzed by an Eksigent NanoLC system (SCIEX). The solution was resolved on a C18 capture column (5 μm, 100 μm×20 mm), and gradient elution was performed on a C18 analytical column (3 μm, 75 μm×150 mm) with a 90 min time gradient at a flow rate of 300 nL/min. For information-dependent acquisition (IDA), the MS spectrum was detected with an ion accumulation time of 250 ms. Later, the MS spectrum with 30 precursor ions was accessed with an ion accumulation time was 50 ms. Furthermore, the MS1 spectrum was collected in the range of 350-1500 m/z, and the MS2 spectrum was collected in the range of 100-1500 m/z. The precursor ion dynamic exclusion time was set to 15 s.

We used the search engine matched with AB Sciex 5600 plus-ProteinPilot™ (V4.5), which considered all possible modification types, and added an automatic fault-tolerant matching function. For the identified proteins, we considered an Unused ProtScore ≥ 1.3 (the reliability level was above 95%), and each protein contained at least one unique peptide as a trusted protein [15, 16].

### Bioinformatics analysis

All identified proteins were evaluated by a Gene Ontology analysis. The cellular components, biological processes, and molecular functions were identified by searching the PANTHER database (http://www.pantherdb.org/).

### Western blot

The cells were collected and lysed after 48 h of transfection. The cell suspension was separated by centrifugation at 12000 rpm for 5 min, and the supernatant was pooled. Protein concentration was measured by a BCA kit. Proteins were separated by SDS-PAGE (10%) and transferred to PVDF membranes. Then the membranes were incubated with primary and secondary antibodies. Protein bands were exposed via a ChemiDoc MP imaging system (Bio-Rad Laboratories, Hercules, CA, USA).

### Immunofluorescence (IF)

The cells were fixed with 4% paraformaldehyde for 10 min and washed with PBS. Cell membranes were ruptured using 0.2% Triton X-100 and the cells were washed with PBS an additional 2 times. The cells were incubated with primary antibodies overnight at 4° C, rewashed with PBS 3 times, and incubated with secondary antibodies at 37° C in the dark for 1 h. The cells were washed again with PBS in the dark, the cell nuclei stained with DAPI, and images were taken with a confocal laser scanning microscopy (Nikon Corporation, Tokyo, Japan) at 400x magnification.

### RT-qPCR

Total RNA was isolated from HepG2 cells with TRIzol reagent (Invitrogen; Thermo Fisher Scientific, Inc.), according to the manufacturer’s protocol. Subsequently, cDNA was synthesized using a Reverse Transcription kit (Thermo Fisher Scientific, Inc.). Quantitative real-time PCR was subsequently performed with SYBR Premix Ex Taq (Takara, Dalian, China) using an ABI 7500 instrument (Applied Biosystems Inc). The products were analyzed by the 2^−ΔΔCt^ method [17].

### Statistical analysis

All experiments were conducted at least three times, and quantitative variables were presented as the mean ± standard deviation (SD). The statistical analysis and graph illustration were performed using GraphPad Prism v8.0 (GraphPad Software, La Jolla, CA, USA). Student’s t-test or a Mann–Whitney U test was used to analyze data between groups. In addition, the χ^2^ test was applied for qualitative variables. Statistical significance was defined as *p*-value <0.05 for differences between the experimental and control groups. “*” implies a *p*-value <0.05, “**” implies a *p*-value <0.01, “***” implies a *p*-value <0.001 and “****” implies a *p*-value <0.0001; these symbols are marked above the histograms.

### Data availability statements

The data generated in the present study may be requested from the corresponding author.

## RESULTS

### Overexpression of ALT1 in HCC tissues

**Figure 1:**
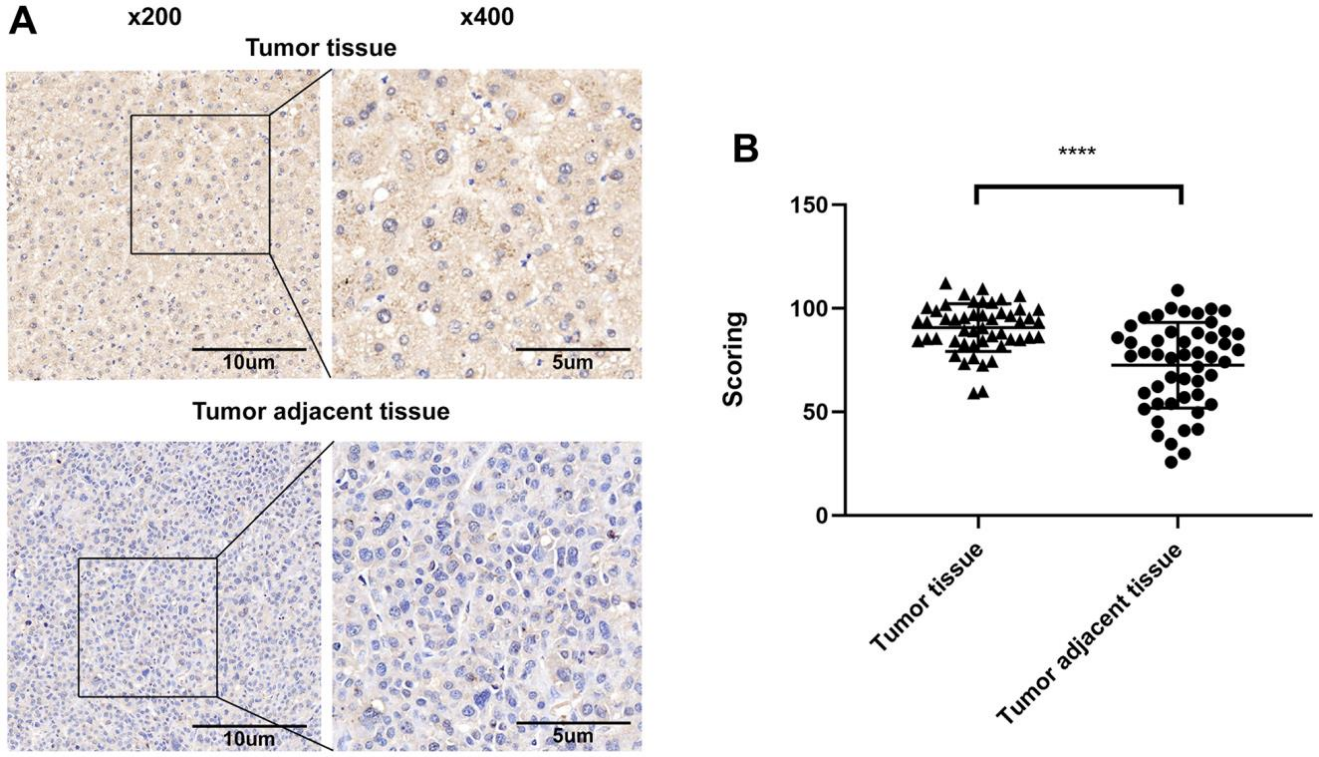
(**A**) Representative images of the immunohistochemical analysis of ALT1 in HCC tissue and matched non-tumor adjacent tissues. (**B**) IHC score values of ALT1 are significantly higher in HCC tissues compared to matched non-tumor tissues.

The expression of ALT1 in tissue microarrays containing 50 cases of HCC tissues and 50 cases of matched non-tumor adjacent tissues was evaluated via IHC. Darker staining occurred in the HCC samples, while lighter staining occurred in the non-tumor adjacent tissues (Figure 1A). The assessment of IHC scores demonstrated higher expression of ALT1 in HCC tissues (*p*<0.05) (Figure 1B).

### Expression of ALT1 in cell lines

**Figure 2:**
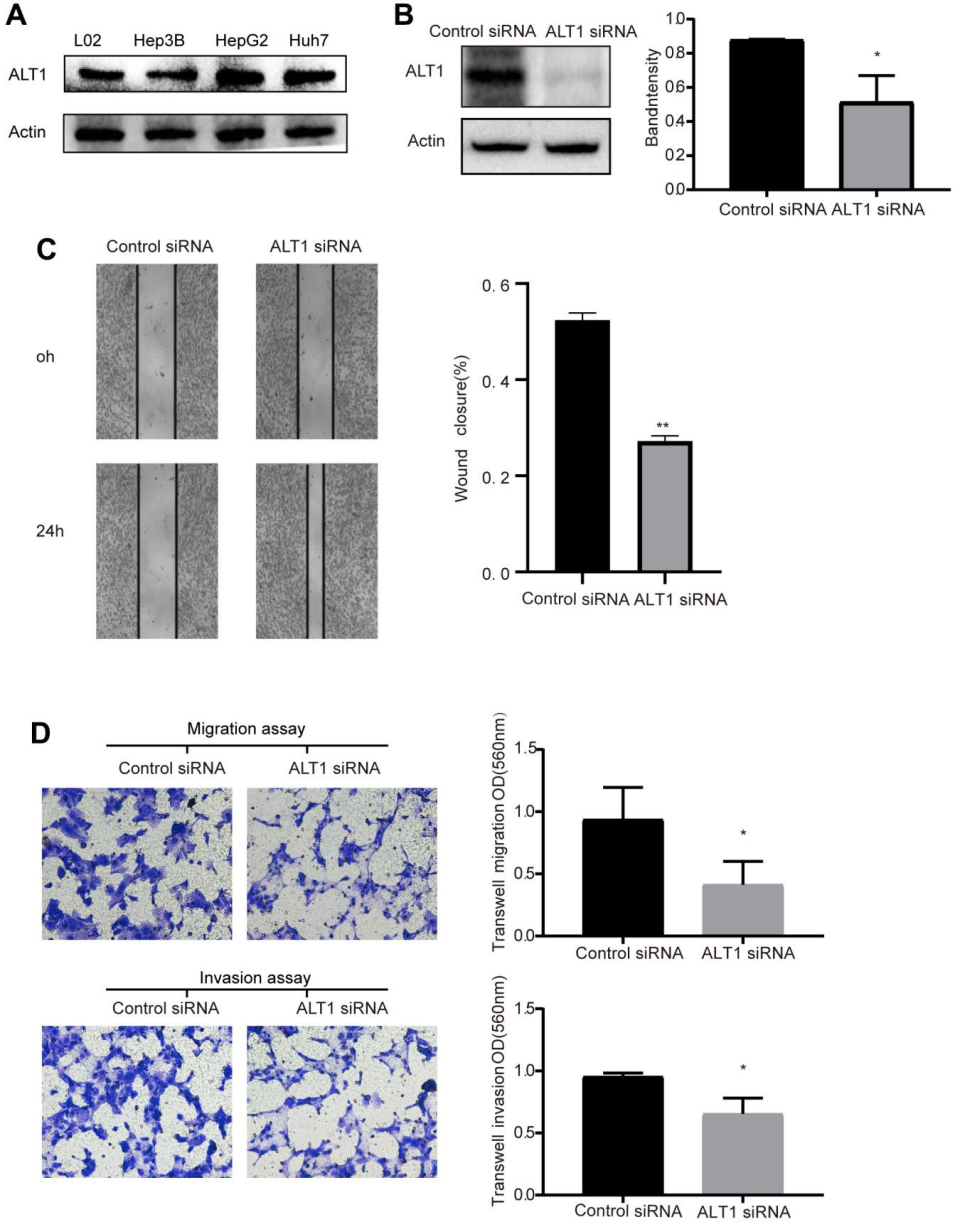
(**A**) Western blot analysis of ALT1expression in L02, Hep3B, HepG2, and Huh7 cells. (**B**) Quantitative analysis results and representative images of the Western blot results for ALT1. (**C**) The migration ability of cells in the wound healing assays following ALT1 silencing (10× magnification). (**D**) The migration and invasion ability of cells in the transwell assays following ALT1 silencing.

We conducted western blot analysis with un-transfected L02 (normal liver cell) and liver tumor cell lines Hep3B, HepG2, and Huh7. The expression of ALT1 in HepG2 cells was not only higher than in the other liver cancer cell lines, but also greater than in L02 (Figure 2A). Therefore, the HepG2 cells were utilized for subsequent experiments.

### Effects of ALT1 knockdown on the migration and invasion of HepG2 cells

Wound healing and transwell assays were performed to assess the effects of ALT1-knockdown on the migration and invasion of HepG2 cells. ALT1 specific siRNA suppressed the ALT1 expression (Figure 2B). Second, the results of the wound healing assay demonstrated that ALT1-knockdown significantly inhibited the migration of HepG2 cells compared with the negative control group (Figure 2C). Additionally, we also used a transwell assay to measure the migration and invasion ability of HepG2 cells. The OD values indicated that the migration and invasion capabilities of HepG2 cells were significantly decreased by suppressing ALT1 (Figure 2D).

### Effects of ALT1-knockdown on proliferation and apoptosis of HepG2 cells

**Figure 3:**
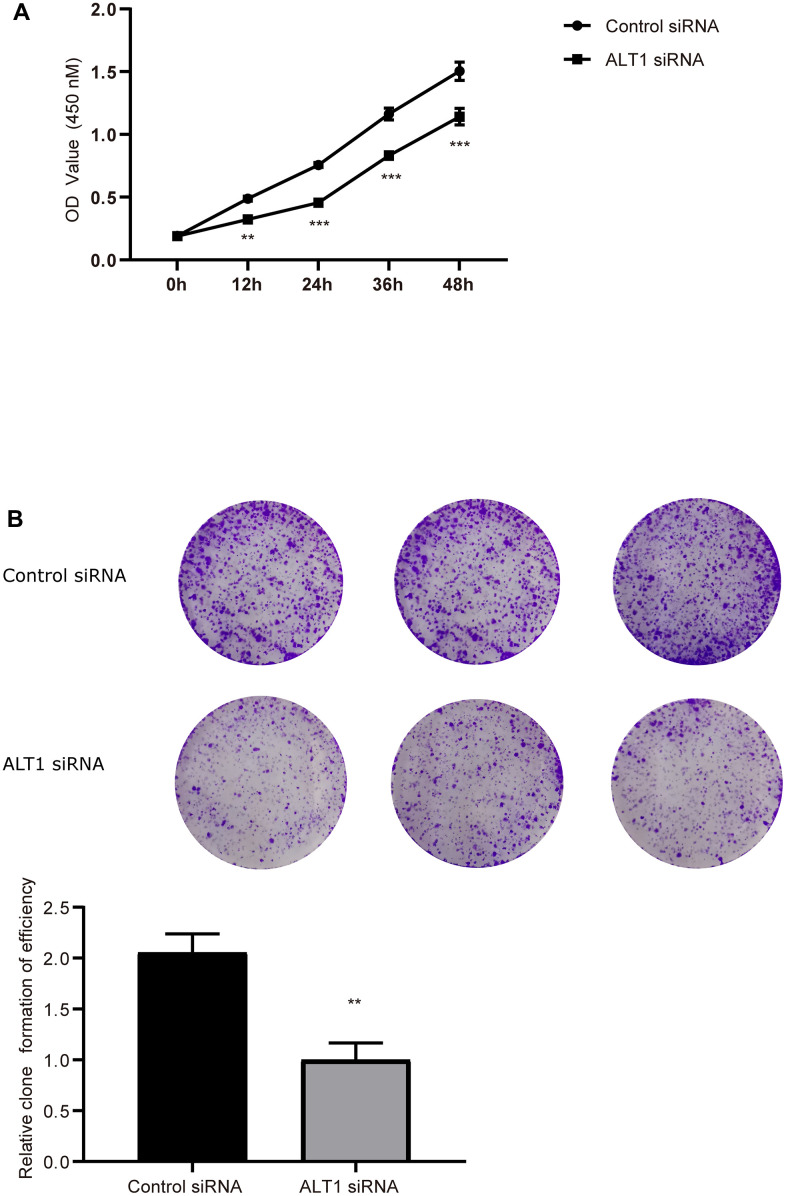
(**A**) Quantitative analysis results of the CCK-8 assay of HepG2 cell. (**B**) Colony formation analysis of HepG2 cell proliferation.

**Figure 4:**
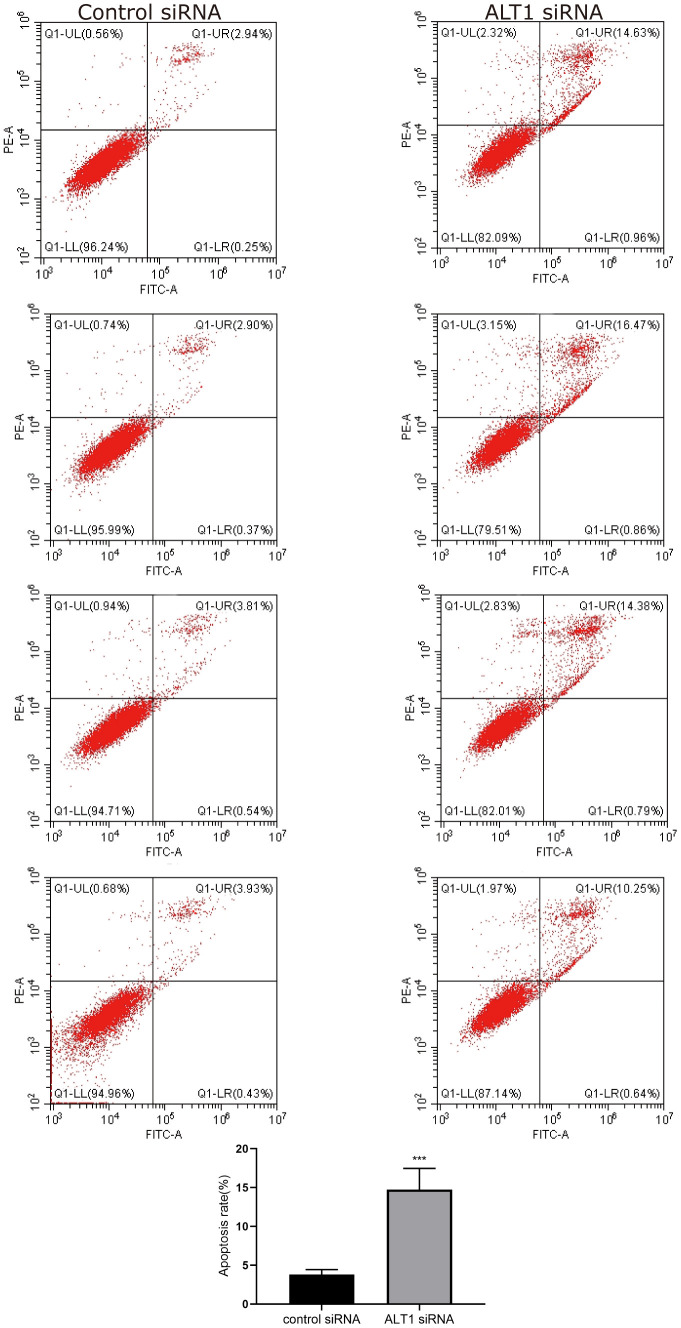
Flow cytometry images and quantitative analysis results of HepG2 cell apoptosis.

**Figure 5:**
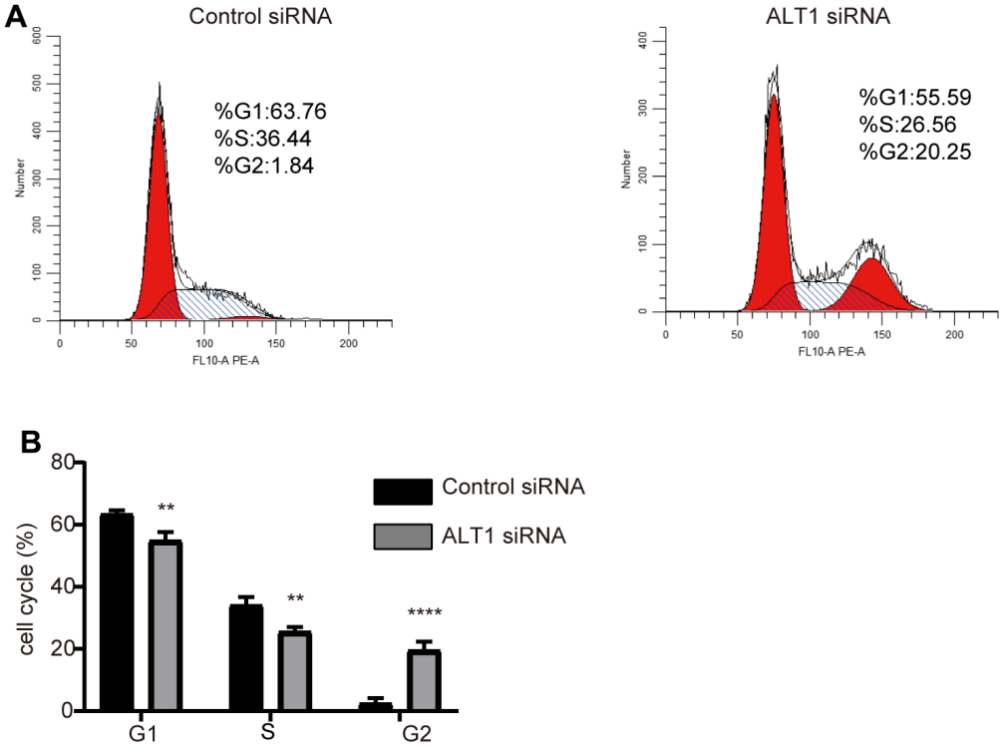
(**A**) Flow cytometry images of the cell cycle distribution in the negative control and ALT1 knockdown cells. (**B**) Quantitative analysis results of the cell cycle distribution in the negative control and ALT1 knockdown cells.

The CCK-8 and colony formation assays showed vast differences between the ALT1-knockdown group and the negative control group, which implied that the proliferation of HepG2 cells was inhibited by suppressing ALT1 (Figure 3A, 3B). Additionally, flow cytometry suggested that the apoptosis rate of the ALT1-knockdown group was remarkably greater than that of the control group (Figure 4). The cell cycle analysis demonstrated the number of cells in G1 and S phases decreased, while the number of cells in G2 phase increased significantly, suggesting that cell arrest occurred in G2 phase (Figure 5).

### iTRAQ quantification of the ALT1 interactome

**Figure 6:**
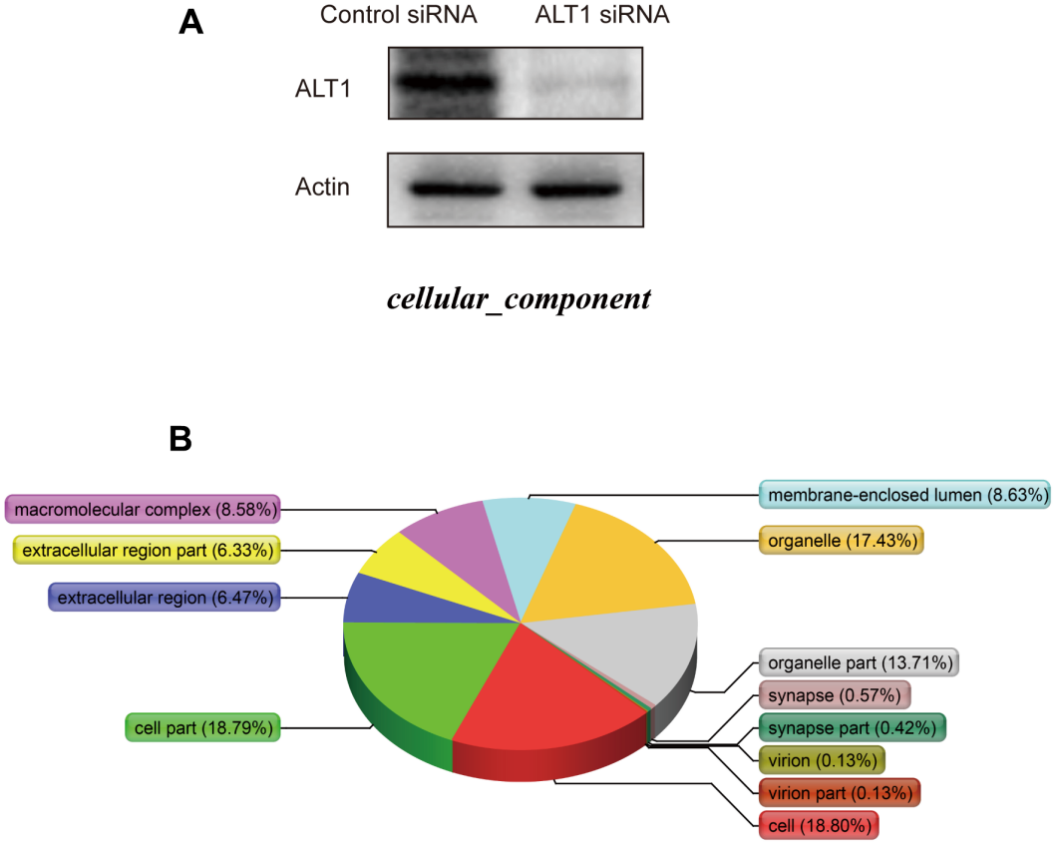
(**A**) Western blot analysis of ALT1 knockdown efficacy before iTRAQ detection. (**B**) Cellular components.

Supplementary Figure 1 details the process of iTRAQ labeling and detection. Before iTRAQ-based MS detection, the knockdown efficiency of ALT1 was analyzed, and the results showed suppressed expression of ALT1 as compared to that in the control group (Figure 6A). A total of 116 DEPs were identified, consisting of 49 upregulated DEPs and 67 downregulated DEPs (Supplementary Table 1).

### DAVID analysis of ALT1-interacting proteins

**Figure 7:**
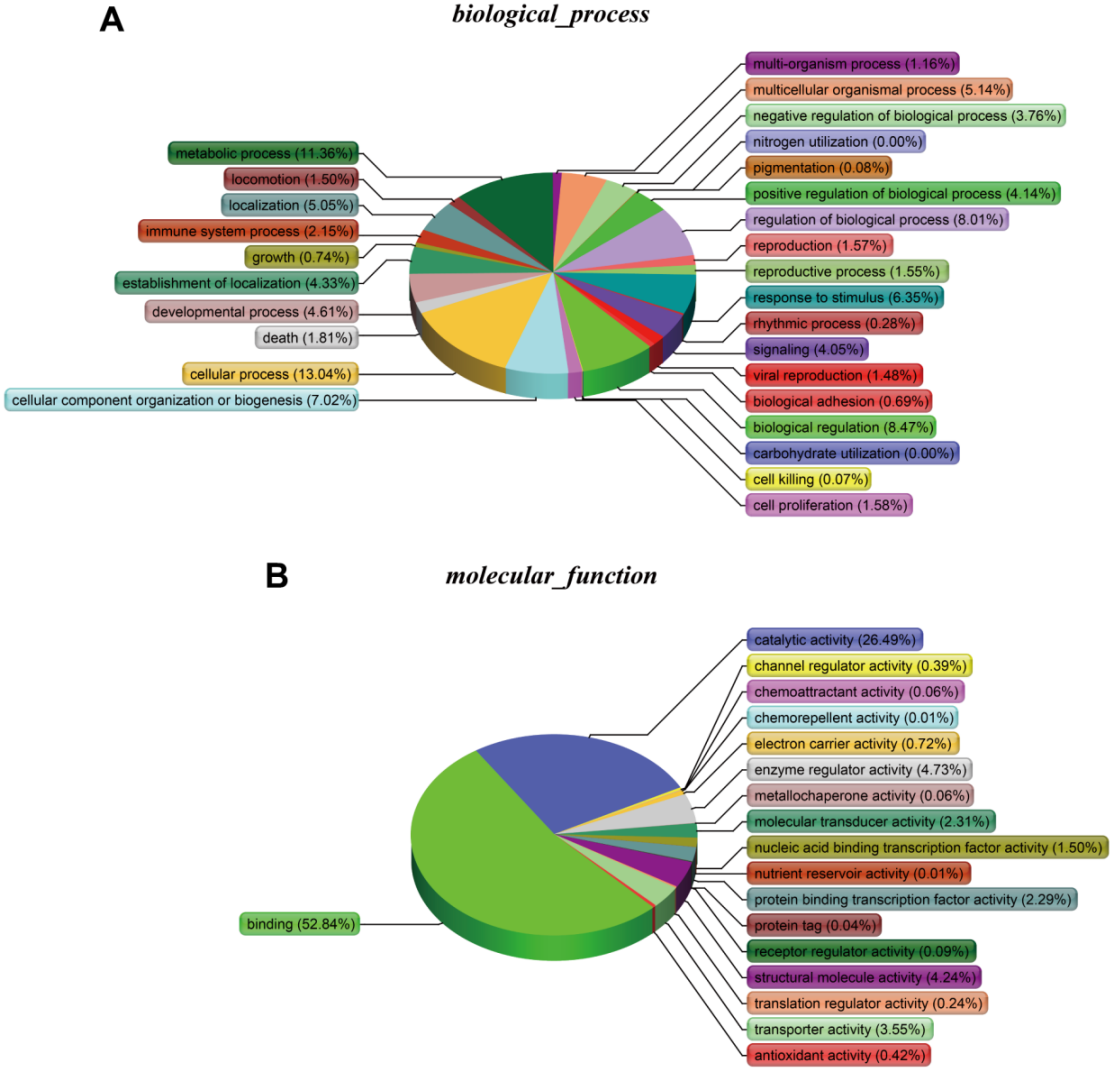
(**A**) Biological processes. (**B**) Molecular functions.

The cellular components (Figure 6B), biological processes (Figure 7A), and molecular functions (Figure 7B) were identified via PANTHER. Among the cellular components, the cell occupied the maximal portion with 18.8%, while the cell part ranked second at 18.79%. Among the biological processes, cellular processes accounted for the largest proportion (13.04%), followed by metabolic processes (11.36%). The molecular function analysis involved mainly binding (52.84%) and catalytic activity (26.49%).

### Validation of iTRAQ outcomes

**Figure 8:**
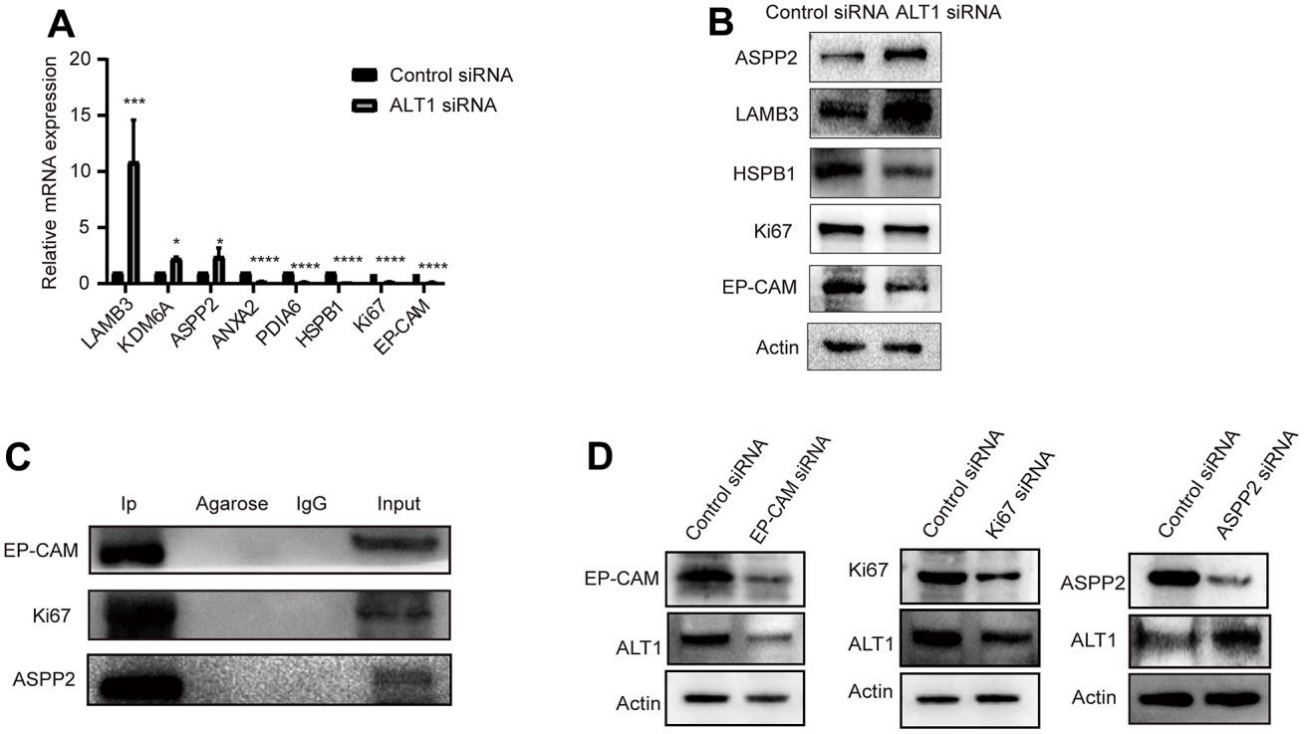
(**A**) RT-qPCR measured the relative mRNA expression levels of LAMB3, KDM6A, ASPP2, ANXA2, PDIA6, HSPB1, KI67, and EP-CAM. (**B**) A representative Western blot analysis for ASPP2, LAMB3, HSPB1, KI67, and EP-CAM expression in HepG2 cells. (**C**) A representative Western blot analysis for EP-CAM, KI67, and ASPP2 co-immunoprecipitation with ALT1 used as a bait protein. (**D**) A representative Western blot analysis for ALT1 expression in EP-CAM, KI67, and ASPP2 knockdown HepG2 cells.

Eight proteins from the 116 DEPs were selected based on the iTRAQ results. The mRNA expression levels of ASPP2, LAMB3 (laminin subunit beta-3), HSPB1 (heat shock protein beta-1), KDM6A (lysine-specific demethylase 6A), annexin A2, PDIA6 (protein disulfide-isomerase A6), EP-CAM, and Ki67 were detected by RT-qPCR (Figure 8A). Western blotting was used to evaluate the expression of ASPP2, LAMB3, HSPB1, EP-CAM, and Ki67 (Figure 8B). The RT-qPCR and Western blot outcomes were both consistent with those of iTRAQ.

### Validation of the interaction between EP-CAM, KI67, ASPP2, and ALT1

**Figure 9:**
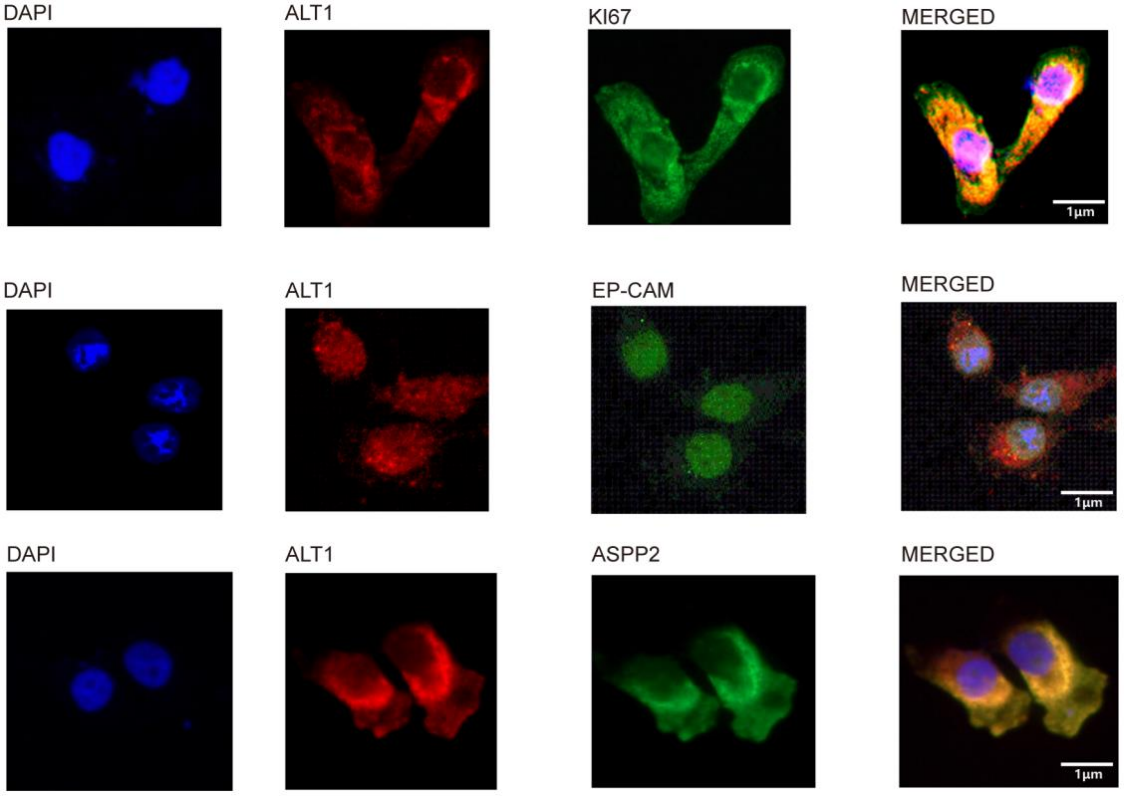
Co-localization of ALT1 and ALT1-binding proteins.

Three main DEPs were selected for evaluation further. EP-CAM, KI67, and ASPP2 were captured by co-IP with ALT1 used as a bait protein, indicating a direct interaction between ALT1 and these three DEPs. (Figure 8C) Knockdown of EP-CAM and KI67 decreased the expression of ALT1, while knockdown of ASPP2 increased ALT1 expression. (Figure 8D). Confocal microscopy was used to determine the subcellular co-location of these three key DEPs with ALT1 (Figure 9).

### Effects of ALT1 and EP-CAM knockdown on MMPs and EMT in HCC

**Figure 10:**
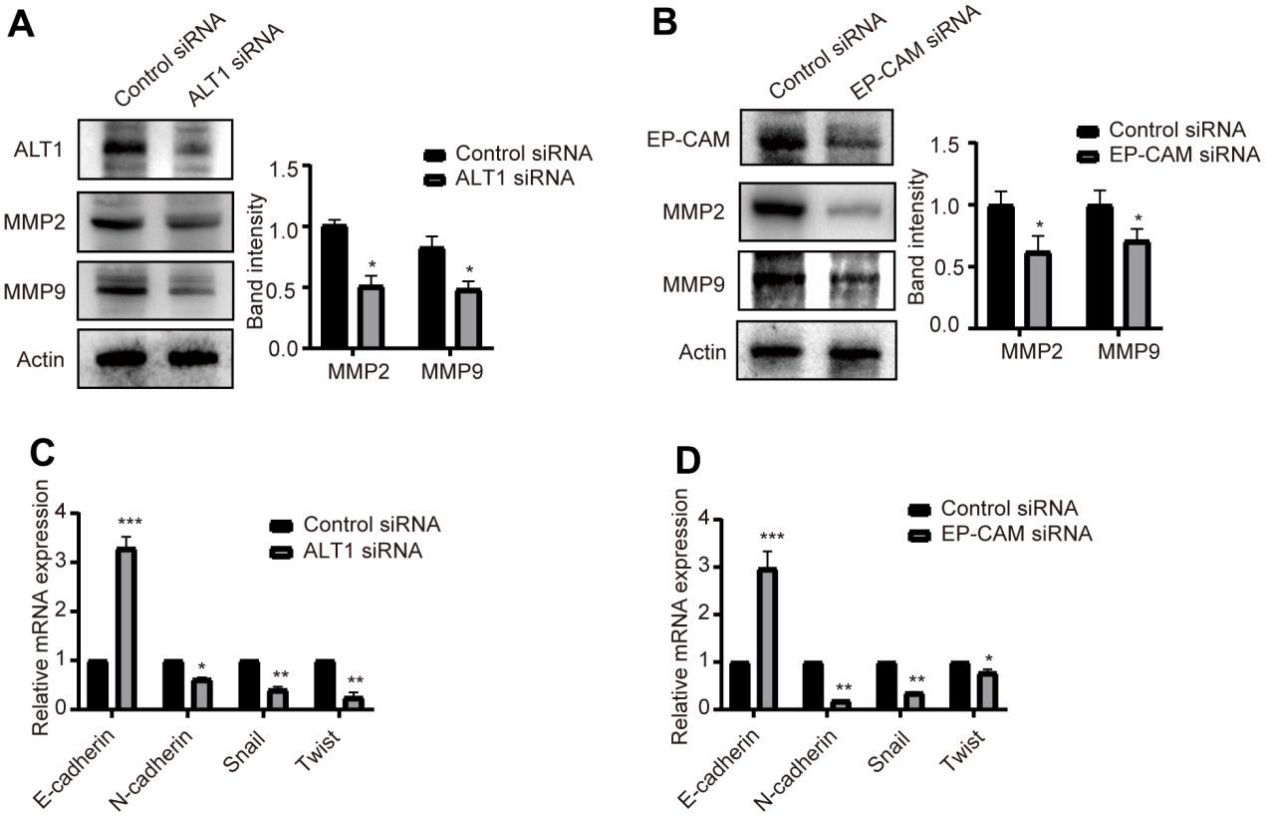
(**A**) Quantitative analysis results and representative images of the Western blot results for MMP2 and MMP9 in ALT1 knockdown HepG2 cells. (**B**) Quantitative analysis results and representative images of the Western blot results for MMP2 and MMP9 in EP-CAM knockdown HepG2 cells. (**C**) RT-qPCR analyses showed the knockdown of ALT1 in HepG2 cells significantly decreased expression of N-cadherin, Snail, and Twist while increasing E-cadherin expression. (**D**) RT-qPCR analyses showed the knockdown of EP-CAM in HepG2 cells significantly decreased expression of N-cadherin, Snail, and Twist while increasing E-cadherin expression.

Alterations in MMP levels and EMT are closely related to the migration and invasion of cancers. Western blot detection showed that the expression of MMP2 and MMP9 was suppressed when ALT1 and EP-CAM were knocked down (Figure 10A). Furthermore, RT-qPCR was used to verify the EMT-related markers E-cadherin, N-cadherin, Snail, and Twist. Despite increased expression of the E-cadherin mRNA, the expression of N-cadherin, Snail, and Twist was decreased. (Figure 10B) These results suggested that after ALT1 interacted with EP-CAM, it participated in the migration and invasion of HepG2 cells by influencing EMT and MMPs.

### Effects of ALT1 and KI67 knockdown on the expression of markers of proliferation and apoptosis

**Figure 11:**
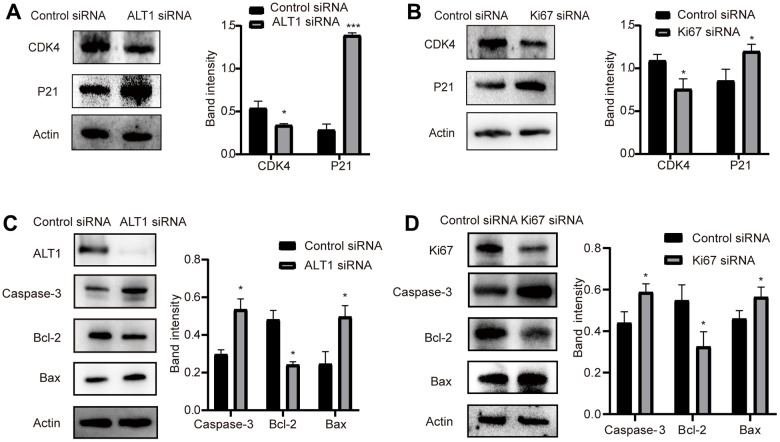
(**A**) Quantitative analysis results and representative images of the Western blot results for cleaved CDK4 and P21 in ALT1 knockdown HepG2 cells. (**B**) Quantitative analysis results and representative images of the Western blot results for cleaved CDK4 and P21 in Ki67 knockdown HepG2 cells. (**C**) Quantitative analysis results and representative images of the Western blot results for cleaved caspase-3, Bax, and Bcl-2 in ALT1 knockdown HepG2 cells. (**D**) Quantitative analysis results and representative images of the Western blot results for cleaved caspase-3, Bax, and Bcl-2 in Ki67 knockdown HepG2 cells.

Cleaved caspase 3, Bax, and Bcl-2 are common markers associated with apoptosis, and P21 and CDK4 are common markers related to proliferation. When the expression of ALT1 and KI67 was inhibited, the expression of Bcl-2 and CDK4 decreased, while the expression of cleaved caspase 3, Bax, and P21 increased (Figure 11A, 11B). These findings suggest that ALT1 interacts with KI67 to participate in the proliferation and apoptosis of HepG2 cells.

### Effects of ALT1 and ASPP2 knockdown on the P53 signaling pathway

**Figure 12:**
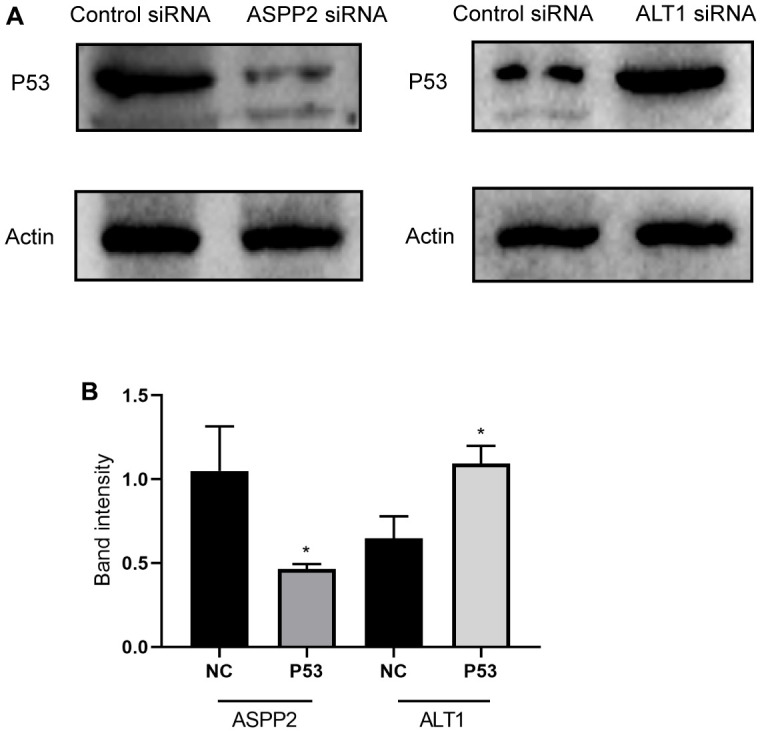
(**A**) Representative images of the Western blot results for P53 in ASPP2 and ALT1 knockdown HepG2 cells. (**B**) Quantitative analysis results of the Western blot of P53 expression.

The expression of ASPP2 activates P53 expression in many cancers, and the inhibition of ASPP2 suppresses the expression of P53. Based on the previous Western blot analysis, ALT1 knockdown upregulated the expression of ASPP2. We further inhibited ALT1 and found that the expression of P53 increased (Figure 12A). Figure 12B shows the band intensity of P53 and control group expressions of ASPP2 and ALT1. The results suggested that ALT1 knockdown suppressed the progression and development of HepG2 cells by activating the P53 signaling pathway.

## DISCUSSION

Previous research has suggested that enrichment of ALT2 in the breast can promote the development of breast cancer [8]. On the basis of such hypothesis, we analyzed the expression of ALT1 and we found high expression levels in HCC tissues. However, few studies have reported its effects in HCC cells. When ALT1 expression was inhibited, the migration and invasion capabilities of HepG2 cells decreased. The CCK-8, colony formation, and flow cytometry assays suggested that ALT1 knockdown decreased proliferation and increased apoptosis in HepG2 cells. CO-IP and iTRAQ-based MS identified several DEPs that interacted with ALT1. Based on the Western blot and RT-qPCR analyses, we concluded that ALT1 influenced EMT and MMPs by interacting with EP-CAM. We also concluded that ALT1 was involved in proliferation and apoptosis by interacting with Ki67. Finally, according to the ALT1 interacting protein ASPP2, which plays the role of a P53 activator, we suggested that knockdown of ALT1 suppressed the progression and development of HCC via the P53 signaling pathway.

EP-CAM is a 35-kDa molecule with the capability of transmembrane glycoprotein cell adhesion, which is associated with the adhesion of Ca^2+^-independent adhesion between cells [18, 19]. Additionally, EP-CAM has been applied in the detection of circulating tumor cells [20, 21]. Previous studies have suggested that EP-CAM is associated with cell migration, invasion, signal transduction, and differentiation [22, 23]. The overexpression of EP-CAM in breast cancer, ovarian cancer, and head and neck squamous cell cancer has shown a negative impact on cancer prognosis [24–26]. EP-CAM can strengthen the tumor-initiating ability by influencing EMT, which is induced through N-glycosylation of EpCAM in breast cancer [27–29]. Furthermore, EP-CAM can regulate MMP2 and MMP9 in gastric cancer by activating the NFκB signaling pathway [30]. Based on the above studies, we selected EP-CAM as the key DEP to determine whether ALT1 was involved in migration and invasion. KI67, a 380 kDa nuclear protein, is detected throughout the three phases of the cell cycle. Therefore, the expression of KI67 suggests the proliferation stage of cells [31, 32]. Additionally, KI67 is overexpressed in many cancers and is considered a prognostic marker of cancer [33–35]. ASPP2, as a haploinsufficient tumor suppressor and a member of the p53 activator, stimulated apoptosis with its C-terminus binding with P53 [36, 37]. The P53 signaling pathway is involved in many human cancers, but an increasing number of cancers have evolved to inactivate this common tumor suppressor pathway [38].

EMT is not only a vital biological process that is activated through the c-met signaling pathway but also an essential initiation step in tumor migration and invasion [39]. In addition, MMPs are a series of major proteolytic enzymes, and they can regulate tumor metastasis by decomposing the extracellular matrix [39]. In this study, silencing ALT1 and EP-CAM expression in HepG2 cells contributed to alterations in the mRNA expression of EMT-related biomarkers, including E-cadherin, N-cadherin, Snail, and Twist, and changes in MMP2 and MMP9. However, some limitations remain. First, Western blotting identified the expression of ALT1, but the iTRAQ analysis did not detect it, which may have been caused by the low abundance of ALT1 before peptide detection. The results of the Western blot and RT-qPCR analyses of DEPs interacting with ALT1 proved the reliability of the iTRAQ screening results. Additionally, few liver cancer cell lines were included in the current study. Although HepG2 cells are most commonly used in drug metabolism and hepatotoxicity studies, coupled with their higher proliferation rates than other liver cell lines, further studies are needed to examine the effect of ALT1 inhibition in other liver cell lines [40]. Finally, more research is required to study further how ALT1 interacts with the DEPs identified via iTRAQ.

## CONCLUSIONS

In conclusion, iTRAQ proteomics analysis identified 116 proteins that interact with ALT1. Three proteins that interact with ALT1 are closely associated with tumor biological behaviors. According to the functions of EP-CAM, KI67, and ASPP2, we concluded that ALT1 may bind with these proteins to regulate migration, invasion, proliferation, and apoptosis via the P53 signaling pathway in HCC and may influence EMT and MMPs to alter migration and invasion. However, its potential mechanism in proliferation and apoptosis remains unknown and requires further study.

## Supplementary Material

Supplementary Figure 1

Supplementary Table 1
