# Supplementary figures and images for: Identification of alanine aminotransferase 1 interaction network via iTRAQ-based proteomics in alternating migration, invasion, proliferation and apoptosis of HepG2 cells
Source: Aging (Albany NY). 2022 Sep 14;14(17):7137–55. doi: 10.18632/aging.204286 (PMC9512495; doi:10.18632/aging.204286)

## SUPPLEMENTARY FIGURE

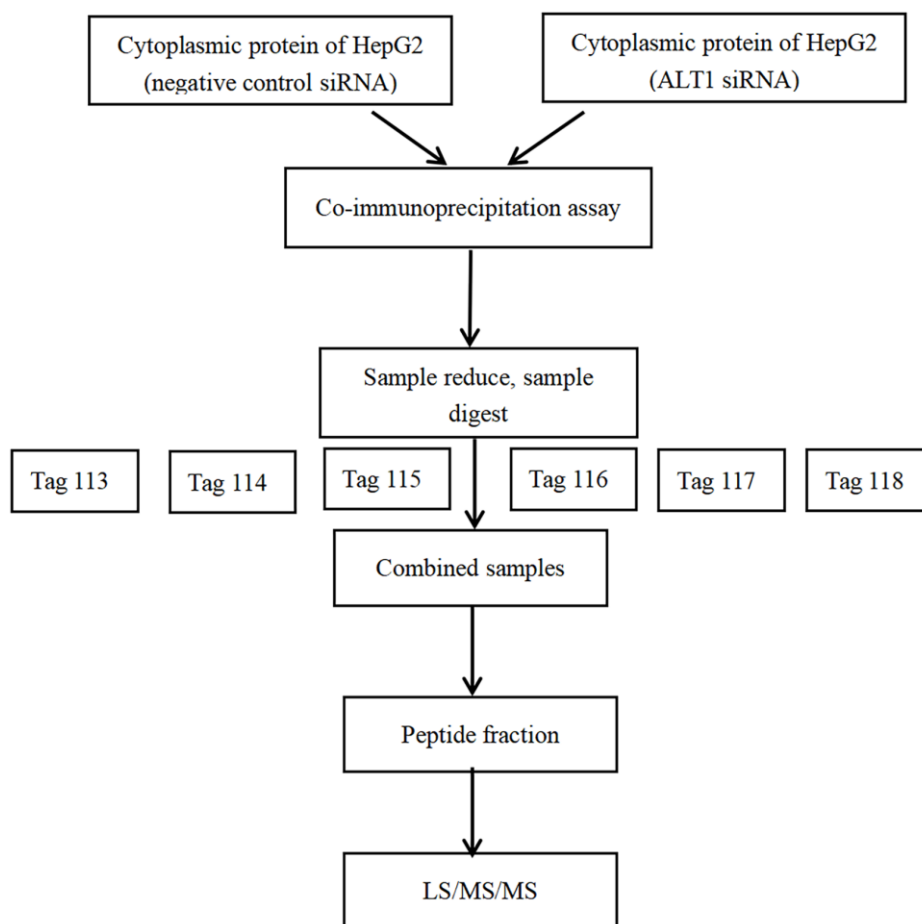

Supplementary Figure 1. The iTRAQ-based MS workflow.

Supplement: Supplementary Figure 1 [file aging-14-204286-s001.pdf]
