# Supplementary material for: Identification of alanine aminotransferase 1 interaction network via iTRAQ-based proteomics in alternating migration, invasion, proliferation and apoptosis of HepG2 cells
Source: Aging (Albany NY). 2022 Sep 14;14(17):7137–55. doi: 10.18632/aging.204286 (PMC9512495; doi:10.18632/aging.204286)
Supplement: Supplementary Table 1 [file aging-14-204286-s002.docx]

**Supplementary Table 1. List of differentially expressed proteins, as identified using iTRAQ technology.**

| **Accession** | **Name** | **Diff_state** | **N:A** | **PVal N:A** |
| --- | --- | --- | --- | --- |
| sp\|P07814\|SYEP_HUMAN | Bifunctional glutamate/proline--tRNA ligase | down | 0.792803879 | 0.01087022 |
| sp\|P05783\|K1C18_HUMAN | Keratin, type I cytoskeletal 18 | down | 0.742946587 | 0.000353783 |
| sp\|P41252\|SYIC_HUMAN | Isoleucine--tRNA ligase, cytoplasmic | down | 0.821523054 | 0.009768226 |
| sp\|O00159\|MYO1C_HUMAN | Unconventional myosin-Ic | down | 0.734331835 | 0.020638241 |
| sp\|P22102\|PUR2_HUMAN | Trifunctional purine biosynthetic protein adenosine-3 | down | 0.769792022 | 0.047847841 |
| sp\|P54577\|SYYC_HUMAN | Tyrosine--tRNA ligase, cytoplasmic | down | 0.646177966 | 0.002156867 |
| sp\|P26641\|EF1G_HUMAN | Elongation factor 1-gamma | down | 0.787852134 | 0.049936108 |
| sp\|P17174\|AATC_HUMAN | Aspartate aminotransferase, cytoplasmic | down | 0.635767105 | 0.009885369 |
| sp\|P05091\|ALDH2_HUMAN | Aldehyde dehydrogenase, mitochondrial | down | 0.783230995 | 0.01297472 |
| sp\|O43175\|SERA_HUMAN | D-3-phosphoglycerate dehydrogenase | down | 0.592603538 | 0.003545604 |
| sp\|P08243\|ASNS_HUMAN | Asparagine synthetase [glutamine-hydrolyzing] | down | 0.569436597 | 0.000674352 |
| sp\|Q9Y262\|EIF3L_HUMAN | Eukaryotic translation initiation factor 3 subunit L | down | 0.804200156 | 0.04667886 |
| sp\|P46060\|RAGP1_HUMAN | Ran GTPase-activating protein 1 | down | 0.799350319 | 0.02796296 |
| sp\|P13798\|ACPH_HUMAN | Acylamino-acid-releasing enzyme | down | 0.740405677 | 0.008923794 |
| sp\|P46781\|RS9_HUMAN | 40S ribosomal protein S9 | down | 0.81225004 | 0.044261429 |
| sp\|A5YKK6\|CNOT1_HUMAN | CCR4-NOT transcription complex subunit 1 | down | 0.724512726 | 0.0147006 |
| sp\|Q16822\|PCKGM_HUMAN | Phosphoenolpyruvate carboxykinase [GTP], mitochondrial | down | 0.642235499 | 0.002520137 |
| sp\|Q9Y617\|SERC_HUMAN | Phosphoserine aminotransferase | down | 0.697747719 | 0.01404908 |
| sp\|P00966\|ASSY_HUMAN | Argininosuccinate synthase | down | 0.669298846 | 0.03787975 |
| sp\|Q14137\|BOP1_HUMAN | Ribosome biogenesis protein BOP1 | down | 0.793751543 | 0.009631023 |
| sp\|P49321\|NASP_HUMAN | Nuclear autoantigenic sperm protein | down | 0.795030344 | 0.028603939 |
| sp\|O95573\|ACSL3_HUMAN | Long-chain-fatty-acid--CoA ligase 3 | down | 0.660341887 | 0.04910947 |
| sp\|Q13247\|SRSF6_HUMAN | Serine/arginine-rich splicing factor 6 | down | 0.816789729 | 0.038277559 |
| sp\|Q14789\|GOGB1_HUMAN | Golgin subfamily B member 1 | down | 0.709280023 | 0.02155439 |
| sp\|Q15050\|RRS1_HUMAN | Ribosome biogenesis regulatory protein homolog | down | 0.715274844 | 0.016473031 |
| sp\|O95163\|ELP1_HUMAN | Elongator complex protein 1 | down | 0.749410326 | 0.032891199 |
| sp\|Q15758\|AAAT_HUMAN | Neutral amino acid transporter B(0) | down | 0.710976371 | 0.0264921 |
| sp\|Q9UJU6\|DBNL_HUMAN | Drebrin-like protein | down | 0.66129683 | 0.02991342 |
| sp\|Q9NRG9\|AAAS_HUMAN | Aladin | down | 0.811203995 | 0.004531072 |
| sp\|O60306\|AQR_HUMAN | RNA helicase aquarius | down | 0.661844472 | 0.002593199 |
| sp\|Q14573\|ITPR3_HUMAN | Inositol 1,4,5-trisphosphate receptor type 3 | down | 0.818897167 | 0.03199479 |
| sp\|P62244\|RS15A_HUMAN | 40S ribosomal protein S15a | down | 0.564510336 | 0.005429749 |
| sp\|O15160\|RPAC1_HUMAN | DNA-directed RNA polymerases I and III subunit RPAC1 | down | 0.740243078 | 0.024419909 |
| sp\|Q9BZE1\|RM37_HUMAN | 39S ribosomal protein L37, mitochondrial | down | 0.713000066 | 0.02531947 |
| sp\|P48634\|PRC2A_HUMAN | Protein PRRC2A | down | 0.640472399 | 0.005960362 |
| sp\|Q99447\|PCY2_HUMAN | Ethanolamine-phosphate cytidylyltransferase | down | 0.799402898 | 0.002745488 |
| sp\|P02768\|ALBU_HUMAN | Serum albumin OS=Homo sapiens | down | 0.483727251 | 0.029203501 |
| sp\|Q86V21\|AACS_HUMAN | Acetoacetyl-CoA synthetase | down | 0.61963473 | 0.01440966 |
| sp\|Q96IU4\|ABHEB_HUMAN | Protein ABHD14B OS=Homo sapiens | down | 0.571668449 | 0.017714869 |
| sp\|Q93096\|TP4A1_HUMAN | Protein tyrosine phosphatase type IVA 1 | down | 0.681451378 | 0.042571779 |
| sp\|Q13884\|SNTB1_HUMAN | Beta-1-syntrophin | down | 0.734371898 | 0.02866016 |
| sp\|Q13459\|MYO9B_HUMAN | Unconventional myosin-IXb | down | 0.524364463 | 0.034658901 |
| sp\|P11117\|PPAL_HUMAN | Lysosomal acid phosphatase | down | 0.756923116 | 0.00245728 |
| sp\|Q99829\|CPNE1_HUMAN | Copine-1 | down | 0.611795564 | 0.033692122 |
| sp\|Q8NBL1\|PGLT1_HUMAN | Protein O-glucosyltransferase 1 | down | 0.746152208 | 0.043779399 |
| sp\|Q13751\|LAMB3_HUMAN | Laminin subunit beta-3 | down | 0.509489846 | 0.027609119 |
| sp\|Q13625\|ASPP2_HUMAN | Apoptosis-stimulating of p53 protein 2 | down | 0.765284522 | 0.025569901 |
| sp\|O15550\|KDM6A_HUMAN | Lysine-specific demethylase 6A | down | 0.803409447 | 0.039863098 |
| sp\|Q8WWQ0\|PHIP_HUMAN | PH-interacting protein | down | 0.592842601 | 0.003504451 |
| sp\|P15924\|DESP_HUMAN | Desmoplakin | up | 1.255141751 | 0.004822595 |
| sp\|Q00610\|CLH1_HUMAN | Clathrin heavy chain 1 | up | 1.422023761 | 0.002099092 |
| sp\|P02545\|LMNA_HUMAN | Prelamin-A/C | up | 1.682500004 | 0.000159908 |
| sp\|P14625\|ENPL_HUMAN | Endoplasmin | up | 1.609501507 | 0.01759797 |
| sp\|P07237\|PDIA1_HUMAN | Protein disulfide-isomerase | up | 1.382496768 | 0.017523 |
| sp\|P08727\|K1C19_HUMAN | Keratin, type I cytoskeletal 19 | up | 1.272834424 | 0.021453589 |
| sp\|P30101\|PDIA3_HUMAN | Protein disulfide-isomerase A3 | up | 1.606847108 | 0.02757204 |
| sp\|P15311\|EZRI_HUMAN | Ezrin | up | 1.605028857 | 0.004648781 |
| sp\|P07355\|ANXA2_HUMAN | Annexin A2 | up | 1.211655899 | 0.03883798 |
| sp\|P00558\|PGK1_HUMAN | Phosphoglycerate kinase 1 | up | 2.412318221 | 0.000216224 |
| sp\|P04075\|ALDOA_HUMAN | Fructose-bisphosphate aldolase A | up | 1.716734348 | 0.006109887 |
| sp\|P19367\|HXK1_HUMAN | Hexokinase-1 | up | 1.707329873 | 0.01684387 |
| sp\|P11388\|TOP2A_HUMAN | DNA topoisomerase 2-alpha | up | 1.225871427 | 0.018189371 |
| sp\|P46013\|KI67_HUMAN | Proliferation marker protein Ki-67 | up | 1.744001152 | 1.0732E-07 |
| sp\|P04181\|OAT_HUMAN | Ornithine aminotransferase, mitochondrial | up | 1.660857244 | 0.005669426 |
| sp\|P00338\|LDHA_HUMAN | L-lactate dehydrogenase A chain | up | 2.196876263 | 0.000803827 |
| sp\|Q9BQG0\|MBB1A_HUMAN | Myb-binding protein 1A | up | 1.229353655 | 0.03885521 |
| sp\|Q02809\|PLOD1_HUMAN | Procollagen-lysine,2-oxoglutarate 5-dioxygenase 1 | up | 1.492765124 | 0.01354179 |
| sp\|P09525\|ANXA4_HUMAN | Annexin A4 | up | 1.401961768 | 0.01017233 |
| sp\|P11717\|MPRI_HUMAN | Cation-independent mannose-6-phosphate receptor | up | 1.214901963 | 0.01168341 |
| sp\|P53992\|SC24C_HUMAN | Protein transport protein Sec24C | up | 1.303625544 | 0.02386662 |
| sp\|P09972\|ALDOC_HUMAN | Fructose-bisphosphate aldolase C | up | 1.54582449 | 0.000591119 |
| sp\|P36776\|LONM_HUMAN | Lon protease homolog, mitochondrial | up | 1.276383769 | 0.0435111 |
| sp\|O75874\|IDHC_HUMAN | Isocitrate dehydrogenase [NADP] cytoplasmic | up | 1.276969741 | 0.029420551 |
| sp\|P67809\|YBOX1_HUMAN | Y-box-binding protein 1 | up | 1.886596705 | 0.000457834 |
| sp\|P21796\|VDAC1_HUMAN | Voltage-dependent anion-selective channel protein 1 | up | 1.252228907 | 0.018743981 |
| sp\|Q15084\|PDIA6_HUMAN | Protein disulfide-isomerase A6 | up | 1.377583702 | 0.039036799 |
| sp\|P13674\|P4HA1_HUMAN | Prolyl 4-hydroxylase subunit alpha-1 | up | 2.115284384 | 1.66983E-05 |
| sp\|Q96RP9\|EFGM_HUMAN | Elongation factor G, mitochondrial | up | 1.237728558 | 0.040380459 |
| sp\|P08758\|ANXA5_HUMAN | Annexin A5 | up | 1.221398691 | 0.0336514 |
| sp\|P49756\|RBM25_HUMAN | RNA-binding protein 25 | up | 1.23339502 | 0.003537194 |
| sp\|Q9ULV4\|COR1C_HUMAN | Coronin-1C | up | 1.233172904 | 0.046842352 |
| sp\|P52789\|HXK2_HUMAN | Hexokinase-2 | up | 1.666776161 | 0.01228437 |
| sp\|Q15021\|CND1_HUMAN | Condensin complex subunit 1 | up | 1.50600265 | 0.039348561 |
| sp\|Q9Y6Y8\|S23IP_HUMAN | SEC23-interacting protein | up | 1.533228845 | 0.02520846 |
| sp\|P11166\|GTR1_HUMAN | Solute carrier family 2, facilitated glucose transporter member 1 | up | 1.863066207 | 0.01466361 |
| sp\|Q14517\|FAT1_HUMAN | Protocadherin Fat 1 | up | 1.258898674 | 0.03228293 |
| sp\|Q86X29\|LSR_HUMAN | Lipolysis-stimulated lipoprotein receptor | up | 1.628985113 | 0.02857751 |
| sp\|P30044\|PRDX5_HUMAN | Peroxiredoxin-5, mitochondrial | up | 2.057733529 | 0.01283083 |
| sp\|Q14108\|SCRB2_HUMAN | Lysosome membrane protein 2 | up | 1.928419914 | 0.039221279 |
| sp\|P07602\|SAP_HUMAN | Prosaposin | up | 1.372000579 | 0.01384656 |
| sp\|O00468\|AGRIN_HUMAN | Agrin | up | 1.62505393 | 0.007491595 |
| sp\|P04792\|HSPB1_HUMAN | Heat shock protein beta-1 | up | 1.345270611 | 0.048009031 |
| sp\|P49821\|NDUV1_HUMAN | NADH dehydrogenase [ubiquinone] flavoprotein 1, mitochondrial | up | 1.405244845 | 0.033441119 |
| sp\|Q92619\|HMHA1_HUMAN | Rho GTPase-activating protein 45 | up | 1.378649415 | 0.03425701 |
| sp\|P26368\|U2AF2_HUMAN | Splicing factor U2AF 65 kDa subunit | up | 1.374401982 | 0.03858006 |
| sp\|P09104\|ENOG_HUMAN | Gamma-enolase | up | 2.403108563 | 0.004701627 |
| sp\|Q9Y376\|CAB39_HUMAN | Calcium-binding protein 39 | up | 1.391294809 | 0.04487009 |
| sp\|O76031\|CLPX_HUMAN | ATP-dependent Clp protease ATP-binding subunit clpX-like, mitochondrial | up | 1.357533348 | 0.04272997 |
| sp\|Q8WUF5\|IASPP_HUMAN | RelA-associated inhibitor | up | 1.447984725 | 0.0375419 |
| sp\|P08648\|ITA5_HUMAN | Integrin alpha-5 | up | 1.659674007 | 0.003267494 |
| sp\|P29353\|SHC1_HUMAN | SHC-transforming protein 1 | up | 1.276030265 | 0.037934508 |
| sp\|P55081\|MFAP1_HUMAN | Microfibrillar-associated protein 1 | up | 1.553013396 | 0.028217761 |
| sp\|P16401\|H15_HUMAN | Histone H1.5 | up | 1.297085016 | 0.04311119 |
| sp\|P61313\|RL15_HUMAN | 60S ribosomal protein L15 | up | 1.273798279 | 0.006427042 |
| sp\|Q8N5K1\|CISD2_HUMAN | CDGSH iron-sulfur domain-containing protein 2 | up | 2.061148984 | 0.02086189 |
| sp\|P51116\|FXR2_HUMAN | Fragile X mental retardation syndrome-related protein 2 | up | 1.29109013 | 0.03455089 |
| sp\|P16422\|EPCAM_HUMAN | Epithelial cell adhesion molecule | up | 1.495689918 | 0.004267967 |
| sp\|Q15654\|TRIP6_HUMAN | Thyroid receptor-interacting protein 6 | up | 2.710941956 | 0.007083457 |
| sp\|Q16222\|UAP1_HUMAN | UDP-N-acetylhexosamine pyrophosphorylase | up | 1.659967301 | 0.04374326 |
| sp\|Q9NRW7\|VPS45_HUMAN | Vacuolar protein sorting-associated protein 45 | up | 1.506609258 | 0.02571601 |
| sp\|O75531\|BAF_HUMAN | Barrier-to-autointegration factor | up | 1.275808056 | 0.02700844 |
| sp\|Q9NRZ9\|HELLS_HUMAN | Lymphoid-specific helicase | up | 1.529224055 | 0.034564011 |
| sp\|Q96K19\|RN170_HUMAN | E3 ubiquitin-protein ligase RNF170 | up | 1.482982635 | 0.03748608 |
| sp\|P49761\|CLK3_HUMAN | Dual specificity protein kinase CLK3 | up | 2.197261426 | 0.02264297 |
| sp\|P10586\|PTPRF_HUMAN | Receptor-type tyrosine-protein phosphatase F | up | 1.222208541 | 0.033641972 |
| sp\|Q8TER5\|ARH40_HUMAN | Rho guanine nucleotide exchange factor 40 | up | 1.70021811 | 0.01299789 |
